# A qualitative exploration of ‘thrivership’ among women who have experienced domestic violence and abuse: Development of a new model

**DOI:** 10.1186/s12905-019-0789-z

**Published:** 2019-08-06

**Authors:** Isobel Heywood, Dana Sammut, Caroline Bradbury-Jones

**Affiliations:** Nursing, Institute of Clinical Sciences, College of Medical and Dental Sciences, University of Birmingham, Edgbaston, Birmingham, B15 2TT UK

**Keywords:** DVA, Domestic abuse, Thrivership, Women’s health

## Abstract

**Background:**

Domestic violence and abuse (DVA) is a serious public health issue, threatening the health of individuals the world over. Whilst DVA can be experienced by both men and women, the majority is still experienced by women; around 30% of women worldwide who have been in a relationship report that they have experienced violence at the hands of their partner, and every week in England and Wales two women are killed by their current or ex-partner.

The purpose of this study was to explore the concept of thrivership with women who have experienced DVA, to contribute to our understandings of what constitutes ‘thriving’ post-abuse, and how women affected can move from surviving to thriving.

**Methods:**

Thirty-seven women took part in this qualitative study which consisted of six focus groups and four in-depth interviews undertaken in one region of the UK in 2018. Data were analysed using a thematic analysis approach. Initial findings were reported back to a group of participants to invite respondent validation and ensure co-production of data.

**Results:**

The process of ‘thrivership’ – moving from surviving to thriving after DVA - is a fluid, non-linear journey of self-discovery featuring three ‘stages’ of victim, survivor, and thriver. Thriving after DVA is characterised by a positive outlook and looking to the future, improved health and well-being, a reclamation of the self, and a new social network. Crucial to ensuring ‘thrivership’ are three key components that we propose as the ‘Thrivership Model’, all of which are underpinned by education and awareness building at different levels: (1) Provision of Safety, (2) Sharing the Story, (3) Social Response.

**Conclusions:**

The study findings provide a new view of thriving post-abuse by women who have lived through it. The proposed Thrivership Model has been developed to illustrate what is required from DVA-services and public health practitioners for the thrivership process to take place, so that more women may be supported towards ‘thriving’ after abuse.

## Background

In 1993 the UN Declaration on the Elimination of Violence against Women recognised the gendered nature of violence stating that

“violence against women is one of the crucial social mechanisms by which women are forced into a subordinate position compared with men” [1].

In all countries, most gender-based violence (GBV) is carried out against women by their – predominantly male – intimate partners, in a domestic setting [2]. Almost a third of women worldwide report that they have experienced a form of physical and/or sexual violence by their partner, and approximately 38% of murders of women globally are committed by their male partner [3]. Men, boys and those who identify as lesbian, gay, bisexual, transgender, and queer (LGBTQ) can also be victims [4] of GBV, though it is widely recognised that the majority is experienced by women and girls [5]. Moreover, women and girls as victims of GBV suffer specific, long-term consequences of gender discrimination [4]. Thus, GBV can be viewed as a structural mechanism used to sustain male dominance [4]; equality between women and men cannot exist when women continue to experience gendered violence [6].

In the UK, the term domestic violence and abuse (DVA) is used more commonly than ‘gendered violence’ or ‘intimate partner violence’, and refers to “any incident or pattern of incidents of controlling, coercive or threatening behaviour, violence or abuse between those aged 16 or over who are or have been intimate partners or family members regardless of gender or sexuality” [7]. Abuse can be psychological, physical (such as slapping, or kicking [8]), sexual (such as forced intercourse [8]), financial, or emotional, and can involve controlling behaviour - designed to make a person subordinate and/or dependent by isolating them from sources of support – and coercive behaviour, which is an act or a pattern of acts of assault, threats, humiliation and intimidation or other abuse that is used to harm, punish, or frighten the victim [7]. In the UK women continue to experience more DVA than men; from March 2017 to March 2018 7.9% of women (1.3 million) and 4.2% of men (695,000) experienced DVA in some form, and since the age of 16 28.9% of women had experienced DVA compared to 13.2% of men [9]. The latest Femicide Census reports that 139 women were killed by men in England and Wales during 2017, with 40% of cases featuring ‘overkilling’; where the force and/or methods used to kill a victim was greater than that required to kill them [10]. Three quarters (76%, 105) of women were killed by a man they knew, and almost half (46%) were killed by a current or former intimate partner [10]. Statistics such as these give us some idea of the prevalence of violence against women, though underreporting continues to be an issue [11].

Gendered violence causes substantial harm to women’s physical, mental, sexual, and reproductive health [12]. The physical injuries, fear and stress associated with DVA can result in chronic health conditions including gastrointestinal issues, cardiac symptoms and gynaecological problems [13]. DVA is also a major cause of poor mental health [13] including depression and anxiety [3]; it is estimated that 13% of suicides and suicide attempts by women in the UK may be attributed to domestic violence [14]. Evidence also suggests that victims often struggle with drug and alcohol misuse, as a mechanism for coping with their experiences [13]. DVA has been identified as an adverse childhood experience (ACE) that has a direct graded relationship with health problems in later life including alcoholism, depression and suicide, ischaemic heart disease, and cancer [15]. It is clear then, that DVA is a serious public health issue that crosses geographical and demographic boundaries [16].

During the late 1980s Gondolf and Fisher developed their ground-breaking “survivor theory” [17], arguing that “battered women” are not “passive victims” but are in fact “help-seekers” who show significant strength in their situation through adopting survivor-tactics, and making attempts to gain help from support services that are unsuccessful due to institutional failure [18]. Instead of women being viewed as victims with “learned helplessness” [17], ‘survivor’ became the dominant terminology in the field. However, Wuest and Merritt-Gray [19] argue that assuming the identity of a survivor may not represent the optimal outcome for healing as the term centralises abuse in the lives of women, despite that no longer being the case; whilst for some ‘survivor’ may feel like a badge of honour, for others it may serve as a constant reminder of past negative experiences. It also fails to explore the more long-term recovery from abuse focusing instead on more immediate freedom [20]. ‘Thrivership’ offers a resolve to these issues; if someone is ‘thriving’ they are “prosperous, growing, or flourishing” [21]. Thus, thriving exceeds the absence of problems to signify vigorous, even superlative health and well-being [22].

Much academic focus on DVA has emphasised the role of ‘inner resources’ of individuals in dealing with stressful situations; resilient people, for example, tend to present a high tolerance of distress, or trauma [23]. Resilience has been found to be a positive personality characteristic that enhances adaptation; individuals can present psychological distress juxtaposed with resilience, indicating that resiliency enables women to survive abuse, though nothing beyond that [24]. Whilst these findings contribute to our knowledge of how women may survive trauma, it does not explore what happens afterwards.

Tedeschi and Calhoun [25] advocate the term ‘post-traumatic growth’ to describe the elevated level of functioning that can be experienced following trauma. ‘Thriving’ they argue, does not connote the existence of threat or the shattering of fundamental schemas [26] whereas ‘post-traumatic growth’ indicates that the individual has not only survived but has experienced important changes beyond the previous status quo [25].

We argue though, that ‘thriving’ and ‘post-traumatic growth’ advocate a similar process; when people are thriving, they are not merely surviving or getting by [27], but rather they are growing on an “upward trajectory” [28], and this growth can be in response to trauma experienced.

Thus, studying the ‘thrivership’ process – how someone moves from ‘surviving’ to ‘thriving’ after trauma - allows us to go further with our exploration of how women recover long-term from DVA, so that we may enable others to achieve the same sense of thriving. Thriving may even invite a more complete paradigmatic shift in the investigation of health [22] through furthering our knowledge of adaptive responses to challenges [29] with “an eye toward enhancing health and well-being” [29].

Studies by Paula Poorman [22], Janette Taylor [30], and Wozniak and Allen [20, 31, 32], provide some insight into ‘thriving’ post-DVA, predominantly as a ‘transformative’ process rather than an outcome. ‘Thriving’ or “survivorship-thriving” [30] is a transformative process that represents more than just a return to ‘normal’ [30]; ‘thriving’ denotes active, positive psychological health [22], led by a type of “life energy” [22] that indicates growth and enhanced functionality. The defining and contributing properties of thriving included individual perceptions, motives, and resources; the nature of the relationship a woman has with adversity; and properties of the environment vis-a-vis interpersonal relationships [22]. There is commonly an element of spirituality to findings or approaches: at the ‘thriver stage’ women feel healed, are no longer defined by the abuse, and take care of their physical, emotional and spiritual self [20, 31, 32].

Beyond this however, available literature offers little about how women can be supported through this process by services or practitioners, and what ‘thriving’ means to women beyond a spiritual or theory-based experience. For example, what are the practical implications of ‘thrivership’? What do women need to thrive? How can public health and DVA professionals provide support and services through which thrivership can be attained? The theory of ‘thrivership’, then, is an emerging field; more research is needed to develop an in-depth understanding of what constitutes thriving post-DVA, and what is needed for the thrivership process to take place, according to those who experience it; hence the importance of our study.

## Methods

This was a qualitative study undertaken in a sub-urban setting of one large urban conurbation in central England. Qualitative focus groups were used in order to obtain an insight into the world as experienced by participants [33]; qualitative methodology - crucially for studies around DVA - ‘gives voice’ to people and enables a rich understanding of a phenomenon that cannot be achieved through numbers [34]. Qualitative focus groups were used to ensure in-depth, co-production of knowledge around ‘thriving’ after DVA according to participants. Interviews were offered as an alternative only for those women who were unable to make focus groups, for their convenience.

### Recruitment

Recruitment began in December 2017 and was completed in March 2018. All participants were recruited through a charitable DVA-service that delivers 10–12-week awareness and empowerment programmes for women affected by domestic abuse in the region. Co-author IH conducted recruitment via several visits to the service over the period of 3 months during which potential participants (service users) were approached and information about the study given. In order to be eligible to be involved in the study, participants had to be currently attending at least one programme at the DVA-service. Letters of invitation were distributed in person to women who expressed an interest in being involved, and some Participant Information Sheets were left with the service so that more potential participants could read them when attending service sessions and contact the research team separately. Following initial recruitment, participant contact details were collated so that they could be contacted regarding convenient focus groups dates by the service facilitators. All those who expressed interest were involved in the study except for two women who initially requested phone interviews but did not respond to phone calls or messages. It is not clear why these women did not respond to our attempts to contact them, but for their safety this was not explored in further detail.

### Participant details

A total of 37 participants were involved in the study. All participants were women who had experienced DVA in some form, were attending at least one of the four programmes offered by the DVA-service, and were all (except for one) no longer living with or intimately involved with the perpetrator; service users are only able to move beyond the principal programme delivered at the DVA-service once they have separated from the perpetrator, for safety and recovery reasons. The one participant who was still living with the perpetrator was attending the principal programme and was working with the facilitators at the organisation to plan a safe exit for her from the relationship. Her data was included in the study as we felt it would add a richness to the data to include women at various stages in their recovery and thrivership journey. When asked, women identified as either ‘survivor’ or ‘thriver’, though data on how many identified as each has not been included due to the fluidity of the thrivership process (see findings) which meant that sometimes participants experienced ‘victim days’. Whilst personal data were not gathered specifically for the study as it was not deemed necessary, it was ascertained during focus groups that women were from a range of socio-economic, professional and ethnic backgrounds, and ages ranged from late-teens to sixties. Participants were attending the service due to experiencing DVA at the hands of a male intimate partner; different forms of violence had been experienced by participants, all of which were referred to during discussions, including psychological, emotional, physical, sexual and financial. Whilst focus groups were collated randomly, all women knew someone in their group due to attending programmes together. Whilst levels of engagement in conversation varied - some individuals dominated conversation and others were quieter - all seemed keen to have their voices heard.

### Ethical considerations

Ethical permission was granted from the University of Birmingham (Grant reference ERN_17–1418). Informed written consent was obtained from all participants. Consent forms were collected for each participant and signed by IH, then stored in a locked cupboard in the university. To protect the identity of the participants, all personal data was anonymised upon transcription of the audio data by replacing women’s names with numbers, and ensuring personal data was omitted. Once data analysis had taken place, all transcripts and recordings were deleted permanently. All participants were provided with the NSPCC and Women’s Aid helpline numbers and encouraged to seek support if needed. They were also made aware that extra support could be sought by the DVA-service they attended, as the service staff were trusted individuals known to them.

### Data collection

The intention was to conduct six focus groups each with six participants. However, the first focus group consisted of ten women, which is larger than most focus group sizes. The women had all chosen to stay on to participate after a service programme session and were keen to be involved. Three other groups consisted of six women, and one had five. Four one-to-one interviews were also conducted. The average duration of a focus group was 2 h (with a halfway break) and interviews on average lasted thirty-five minutes.

All focus groups and interviews (apart from one phone interview) took place at the site of the DVA-service, whose programme sessions take place in a group setting. Participant familiarity with the set-up aimed to provide a space where they felt safe and able to share their experience and listen to other women’s views.

Focus groups and interviews were all conducted by co-author IH, who used an interview schedule designed for the study as a guide (see Supplementary Information). Each discussion began with an ice-breaker activity based on Wozniak and Allen’s [32] work. Participants were asked to share feelings or words associated with the terms ‘victim’, ‘survivor’ and ‘thriver’: this encouraged all participants to get involved from the start in the focus groups, provided a good initial overview of the end-to-end recovery process, and enabled a discussion about recovery stages and their labels. During these activities the participants wrote their responses on post-it notes and added them to a large poster, or IH wrote their responses directly onto a poster. These were analysed alongside transcripts of focus group discussions and interview responses.

The same questions were asked in both focus groups and interviews. A scoping review [35] of previous literature in this area was undertaken to form a framework for discussion topics and the questions in the right-hand column were used flexibly as the basis for the interviews (see Table 1). This also enabled the comparison of participant views with those from previous studies (see discussion), whilst the use of focus groups still allowed for the introduction and exploration of new concepts. Qualitative research papers were selected for the scope if they featured an exploration of the concept of ‘thriving’ post-DVA or used the term in relation to DVA recovery. The focus groups and interviews were audio-recorded using a device owned by the University of Birmingham. Recordings were transcribed on to a laptop protected with a password and anti-virus software.

**Table 1:** Themes and constructs from scoping review

Topic	Details
Shattering silences	Was sharing your story/experience a part of the recovery process? Is it necessary to thrive? Has being around other women with the same experiences been an important part of the journey?
Sense of self	How does a thriver’s sense of themselves differ to a non-thriver?
Mental and physical health	What is someone’s health state if they are thriving?
Outlook on life and looking towards the future	How does this change when thriving?
Spirituality and religion	Have either of these played a part in participants' journeys? Are they necessary to thrive?
Healing through forgiveness	Has forgiveness played a part in participants' journeys, and is it necessary to thrive?
Social activism	Has being active either in the community, socially or politically, been a part of the thrivership journey? Have participants used their experiences to help others; Is this necessary to thrive?
Re-joining the community	Do you need your own social group to thrive? How do social groups change when thriving?
Home and safety	Is a home/safe space needed to thrive? What does this look/feel like?
Internal resources	Are there personal characteristics or resources that enable one to thrive?

### Data analysis

Transcription of the audio data was undertaken by IH; a verbatim account of all verbal and non-verbal utterances [36] was produced in order to keep data true to its original nature. This process also enabled IH to familiarise herself with the data prior to analysis and coding. Data from the ice-breaker activity at the beginning of each focus group was also transcribed into word documents and included in the data analysis. Braun and Clarke’s thematic analysis [36] was used to analyse the focus group discussions and responses to interview questions. In the analysis a systematic process was undertaken to find patterned responses or themes within the narrative data set. Initial analysis was conducted by IH, and then DS and CBJ independently validated the emerging themes by examining the data and contributing their own analytic lens. Initial framing of the discussion topics following the scoping review [35] of previous literature in this area, created a good origin for “identifying, analysing, and reporting patterns (themes) within data” [36]; with further themes and ‘sub-themes’ emerging throughout analysis. Three rounds of coding were undertaken to ensure rigorous analysis. All team members have expertise in public health, nursing and/or qualitative research methods.

Six-steps to thematic analysis provided the guide for data analysis [36], with the following specific processes (1) All transcripts were read repeatedly by the research team members to ensure all were familiar with and had obtained a sense of the breadth and depth of the data (2) Initial code generation was performed by IH. Data were organised into meaningful groups that related to the research question and labelled (3) Initial codes were organised into a table using Microsoft Excel. The team met to discuss, verify and sort initial codes into themes based on code similarities. Visual representations were used to explore relationships between codes within themes (4) The themes were reviewed and revised by the team and organized into a coherent pattern, with sub-themes identified and themes that did not have enough data to support them removed. The team then re-examined the data set as a whole to ensure saturation of the data was reached (5). The themes were named and then defined and refined. The scoping review [35] of previous literature in this area was used as a comparator for themes identified during data analysis to assess for similarities (6). A final report was prepared giving a detailed account of each theme. This was primarily produced by IH, with checks and contributions by DS and CBJ.

To validate the findings of this study and continue the ethos of data co-production, a sixth focus group was conducted once initial data analysis had taken place for a feedback session which ran for 2 hours. The group consisted of eight women, all of whom had attended a previous session. Initial findings were presented via PowerPoint presentation and participants provided feedback, amendments or additions. Participants gave positive feedback during the session, reporting that they agreed with our initial findings. There was significant discussion around use of language, particularly the term ‘victim’ (see findings section below).

### Results

Findings are presented under the key themes derived from the analysis, with italicised words spoken by women supporting these themes with the participant number alongside shown as ‘P [number]’.

### The Thrivership process

All except two women said that ‘victim’, ‘survivor and ‘thriver’ were appropriate titles for the stages of recovery and both ‘survivor’ and ‘thriver’ had positive connotations. Many women said the term ‘victim’ was stigmatised within society, and made them feel *weak* ^(P11, P12, P14, P24)^, thus it was difficult for them to accept initially in recovery. However, as they progressed into the ‘survivor’ phase of their recovery *journey* ^(P25)^, accepting that they had been victims became easier.

The two women who disagreed with the label titles were in the same focus group; P19 said that she *wouldn’t identify with those labels,* with P24 adding *I agree with you… But I think they are states of mind at certain points*. P19 then concluded *It is a mix… It’s like well I’m quite a lot of that but I’m still a bit here and there’s a bit of thriver in me.* The other members of the group agreed with use of the language. A short while later, P24 stated *I’m trying to think if I’ve ever identified as a victim…probably not… to me it feels weak.* This prompted P22 to say *it’s because we don’t know [that we’re a victim at the time]*. The issues raised by these participants were presented to the validation focus group for discussion; all members of the group reported feeling strongly during experiencing their ‘victim’ stages they had – similarly to the two participants – disliked use of the word ‘victim’ because of its negative connotations, and that by the time they had reached the ‘survivor’ and ‘thriver’ stages they were able to accept the term ‘victim’ and recognise that they had indeed been victims in some capacity. This was something also highlighted by participants who identified as thrivers in focus groups 1 and 2. It is perhaps worth noting that both participants who raised concerns regarding the labels were attending the principal programme at the DVA-service at the time (thus were early on in their recovery stage), and one was the participant still living with the perpetrator. Importantly their comments highlight the fluidity and personal nature of the recovery and thrivership process.

During the ‘victim’ stage women said they had *no self-esteem* ^(P4, P16, P32)^, felt *powerless* ^(P11, P12, P17, P19)^, believing that the abuse was their fault and reporting that they felt *confused* ^(P17)^, *helpless* ^(P2, P15, P24)^*, lonely* ^(P10)^ and *isolated* ^(P3, P16, P22, P24)^.

The ‘survivor’ stage was more positive; they had *escaped* ^(P29, 30)^ or were no longer with the abuser and thus experienced *freedom* ^(P24)^, they were *coping* ^(P3, P19, P24)^, recognising their own *strength* ^(P21)^ and feeling *resilient* ^(P22, P23)^, but continued experiencing *guilt* ^(P33, P35)^ and *hardships* ^(P18)^.

By the ‘thriver’ stage, all associated emotions or experiences were positive; women reported that they felt *acceptance* ^(P35)^, were *free* ^(P11)^ and *safe* ^(P17)^, could have *fun*, experienced a *clear-mind* ^(P11, P15)^*, clarity* ^(P17)^*, self-confidence* ^(P18)^ and *growth* ^(P18)^; ultimately women were empowered and in *control of their own life* ^(P17)^.

Participants described a fluid and non-linear recovery pathway from ‘victim’ to ‘thriver’ that is vulnerable to triggers. Some used words such as *spectrum* ^(P17)^ or *spiral* ^(P18)^ to describe the process, and one group used the metaphor of a three-point turn. When thriving they were better equipped to recognise triggers and guide themselves back to a positive mental space.

Participants in two interviews and three focus groups drew parallels between thrivership and the grief and loss process and emphasised the subjective nature of the thrivership process; DVA, they said, takes different forms and *severities* ^(P30)^ - people cope differently. Thus, it was important not to feel pressure to recover by a certain point in time.

P29 *– I found it really difficult to view myself as a victim because… I only have recently accepted that I was abused um and by default it makes you a victim. Yeah, so it’s a hard term to accept… it makes you feel weak…*

P27 *– Victim for me… people around me would say or use the term really lightly like ‘oh she’s playing the victim’…so to hear people’s own associations with that word and then to see it like related to something like this, I never wanted to be called that because I thought people would think that it came with an act…*

P3 *– I’ve been through everything in a year…and it’s very easy to move from being a victim to survivor to thinking that you’re there and thriving and then it’s…so easy for that to be knocked right back to the beginning.*

P31 *– You only became a victim because you went through quite psychologically damaging stuff, yeah? Obviously because you’ve got those scars…things can come along and sort of knock you off your thriver line…And I think it’s like real life. Things come along, life comes along.*

P25 *- It’s not a definite thing in the grief and loss process. Like when I split up with him there was times where…I wanted him and I felt like I needed him…then I realised you’ve got grief, loss, acceptance and they fluctuate as well.*

### Characteristics of thriving after DVA

#### Positive outlook and future plans

Women reported that a thriver has a significantly more positive outlook than someone in the other stages; when someone was thriving, they were aware that emotional ‘dips’ or *victim days* ^(P16)^ were normal and would not last. Thriving was accepting that it is *okay to feel shit* ^(P11)^; a change in outlook due to *acceptance and moving on* ^(P21)^.

P29 *– You know as a thriver that this bad day is not gonna last. I can have this bad day and it’s okay and it’s not gonna last whereas when you’re a victim everything is a bit rubbish.*

P6 *– We’ve also learnt that life is a journey, not just after domestic abuse, for everybody, and there are dips in the road…bereavements...money issues…ill health, whatever.*

Acceptance existed conjointly with the belief that the past *doesn’t define the future* ^(P4)^. Participants in three focus groups said thrivers have a newly discovered ability to dictate their life moving forward, take opportunities and make plans due to new-found freedom.

P25 *– I am a thriver, I don’t need to look back.*

P18 *– Well I’d say someone who’s thriving…they’ve got more of a positive outlook or not even just positive but being able to see further into the future and have a long-term plan whereas when you’re not thriving, you’re just surviving - you’re not looking long-term you’re just looking to get through the here and now.*

P21 *– That’s how I see a thriver…accepting where you are. It’s acceptance and moving on.*

#### Improved health and well-being

Women reported that a thriver’s physical and mental health are significantly improved compared to other stages. During the ‘victim’ stage, many women and their children had experienced long-term chronic health conditions because of the abuse, including digestion issues, asthma and mental health issues:

P6 *– I think health issues…the depression, the IBS, the asthma, is all part of …when you’re a victim.*

Once women had escaped the abuse, their health improved due to them no longer being reliant on coping mechanisms or experiencing *fewer illnesses* ^(P36)^ because the abuse-induced health conditions disappeared or were manageable. Improved *self-care* ^(P14)^ – including exercise - also contributed to improved health for some:

P4 *– I need to get fit, I’ve got myself a mountain bike…I’m gonna start swimming…I’m starting to look after my health now…I’ve got a life to live.*

P17 *– [My health has] completely transformed. I mean for one my physical health, you know I think a lot of my issues still if I’m perfectly honest are about coping mechanisms and dealing with the abuse, so you know I drank heavily, I smoked, you know all of the self-harm things really that you do to cope with what’s gone on.*

Thrivers experienced a positive change in mental state and emotional well-being due to feeling calmer, having a *clear mind* ^(P15, P11)^, and experiencing more balanced emotions. They were better equipped to recognise and address anxiety or stress, particularly compared to previous stages when women felt *mentally incapacitated…in a constant state of confusion* ^(P23)^*.*

P35 *– [I’m] less stressed. You’re more able to relax in your circumstances…while I was in denial, I’d flare up and I’d start shouting an’ stuff, but now… I’ve moved forwards, and now there’s no arguing, no shouting – it’s calmer.*

P18 *– I’d say your mental health improves – you’re more positive, more balanced…the slightest thing doesn’t feel like the end of the world.*

One interviewee and women in three focus groups shared experiences of themselves or their children being misdiagnosed, or unnecessarily diagnosed, with mental health conditions rather than having their symptoms viewed as reactions to abuse. Two women referred to social workers enforcing medical tests via general practitioners for mental health disorders.

P33 *– They’re too quick to put a stamp on it, medicate you out of your eyeballs, because it’s so fast for them to just write a prescription...A lot of the time professionals…they lack so much information about the impact…*

P25 *– We had a lot of problems around social services, they put a lot of blame on me…. my doctor put me on the anxiety tablets, but this was all because of social services that I had to do all of these medical analyses.*

P32 *– I was diagnosed with borderline personality disorder while I was with my ex by the mental health team. And if you read up on it so many women who have been in domestic abuse situations or were abused as children, like it’s commonly known that you get it from being abused as a child… they are jumping to diagnose women with this - what I’ve been told is an incurable mental condition – when really it is just an extreme version of PTSD brought on by what you’ve been through.*

Thriving also featured improved health-related help-seeking behaviours; women were more likely to seek help for illness; go for routine appointments (e.g. dentist); or seek counselling support. Management of health-conditions also improved.

P14 *– …and even if you still have depression, you’re probably taking your medication…you’re probably going to counselling…and probably if you did have any health issues you would go and seek healthcare about it whereas as a victim, you’re probably too scared to go to the doctors…or you just weren’t allowed.*

#### Reclamation of self

In all groups, participants discussed thrivership as a journey of self-discovery featuring significantly improved self-worth, confidence, and self-esteem.

Freedom was a crucial element of ‘thriving’; women delighted in the newly discovered power they now had over their lives, after feeling *powerless* ^(P11, P12)^ as victims. Thriving was being able to do *things for yourself* ^(P14)^ such as re-entering education, getting a job, or being able to express emotions openly. Ultimately, women were *taking power back* ^(P34)^.

P33 *– You know that you’re allowed to be happy. You can cry.*

P17 *– It’s just freedom. That’s what it feels like, I’m free…it’s a sense that I’m back in control of my life and I’m in charge of my destiny which is really empowering.*

P2 *– I’ve got a job I’m starting in a couple of weeks and I wouldn’t have done any of that 15 months ago.*

P32 *– Surviving is you do it, thriving is you enjoy doing it…like you’re not just doing it because ‘well I know if I was a normal functioning human being I should be doing it’ but you’re doing it because you get up and you’re like ‘yeah I’m having a fucking great day, and I’m gonna do that and I’m gonna have fun doing it'.*

New-found freedom and empowerment correlated with a deep and powerful journey of self-discovery. For some, this was a re-birth as they figured out who they would *be happy as* ^(P3)^ and could *reinvent* ^(P4)^ themselves. Others used the opportunity to discover everything they liked and disliked.

P15 *– Self-discovery, like finding out about yourself and who you are again. It’s like reminding yourself who you are.*

P24 *– Oh my god – to make decisions for yourself. Like what food do I like? I mean that took me two years to work out what food do I like, what films do I like, and what music do I like because I just didn’t know before.*

Overwhelmingly, thriving was characterised by a realisation or significant increase in *self-worth, self-love* and *believing in yourself* ^(P11)^. This had a hugely positive impact on women's lives, enabling them to: be more assertive, advocate for themselves or their children (e.g. in court or with social workers), go out in public alone and have more faith in their own capabilities.

P4 *– Confidence, self-esteem…is massively improved when you’re a thriver.*

P2 *– I could quite happily now go and sit with a group of strangers…whereas that would’ve been unheard of before I would never have done that.*

#### Social networks

‘Thrivership’ featured a changed social network for all women, and an end to the *isolation* ^(P24)^ they had experienced previously. Social circles expanded due to re-connecting with friends or family they’d lost contact with, and as they gained confidence, they felt more comfortable meeting new people. *Negative relationships* ^(P11)^ left their lives, particularly when setting healthy boundaries.

P29 – …*My family relationships have changed because they weren’t allowed to get very close to me so it changed all of those relationships and for about 15 years they felt like I was stolen from them, so now we have that relationship back.*

P11 *– …And thriver as well is meeting new friends, it’s having a new family, that I-and it’s a new family of women you can identify with...Because my, like my old friends don’t understand me now. I’ve grown, the person I was two years ago they don’t understand me and they probably wouldn’t like me.*

Women reported that at the ‘thriver’ stage, all their relationships should also be thriving, i.e. more *productive…equal…[and] healthier* ^(P17)^. Two interviewees and all focus groups discussed improved relations with family, friends and children. Several groups referred to how their newly-developed knowledge of the signs of abuse contributed to healthier relationships.

P21 *– Some of my friendships are deeper and more solid because this journey from victim to survivor…there’s only two of them, and they’ve walked that bit with me. I call them my sisters now and they will be with me for life, and I couldn’t have said that when I was a victim…*

P4 *– And my relationship with my children as well there’s been a massive change. I’ve got more patience, I’m more forgiving, I’m more thoughtful, they have a voice now.*

Within the context of these new social networks, women reported that as thrivers they felt *more available to help other people* ^(P36)^, than in the victim stage. For some this took the form of social activism (e.g. volunteering at a women’s centre), though most instead offered informal support to other women. E.g. Passing on new knowledge, information and tools, or referring others to the DVA-service.

P11 *– I worked really hard on myself to believe in myself, and to you know pass the knowledge on to other women and learn to empower other women and that’s what I want, to see other women…grow.*

P29 *– …For me thriving is all about sharing and as a woman empowering another woman…when I’m in my thriver state I am sharing.*

### The conditions for Thrivership

#### Provision of safety

Women in all groups reported that the provision of physical safety was vital in ensuring victims could progress towards ‘thriving’ and typically came via having a *safety bubble* ^(P31)^ - usually their home. Three women mentioned practical elements such as security locks offered by Women’s Aid.

P6 *– When I first came here…there was some charity scheme, to get new front doors that were safe…it was to keep you out of having to go in to refuges really but the police came round and assessed the house…that’s an early one, to make you feel safe, you know to be safe…I think without that it would be harder to thrive.*

P18 *– It’s just having my own space you know and knowing that I’m the only one that has keys to that front door. So, I can lock the door and keep the rest of the world out.*

P14 *– [You need a] safe place to talk and to share, get better.*

*Emotional safety* ^(P30)^ or psychological safety was mentioned in relation to acquiring knowledge of how to protect oneself against future abusers and via building self-worth. Safety was also mentioned in relation to having a ‘safe space’ to share their story. All women reported that to thrive it was vital that an individual was taught the appropriate *knowledge* ^(P28)^ and given *tools* ^(P1, P18)^ to recover, including how to develop internal resources (such as resilience and courage), and assertiveness skills. Participants in all groups said that crucial to the thrivership process is education on perpetrator behaviour including the common signs of abuse and the impact of DVA on victims; this provided empowerment, enabled women to make sense of what they had experienced and ensured they didn’t repeat the cycle of abuse.

P29 *– I feel like I’m safe now because I feel I’ve got the knowledge that I need to protect me.*

P27 *– I feel safer in my own decisions and I can rationalise them a lot better. I’ve never felt physically unsafe, but now I’m more aware like in my head of what’s going on.*

P37 *- You don’t have the strength and courage if you can’t build up your self-esteem, and I thought that was just something that you could innately build yourself but it’s not… you do sometimes need to learn how to be stronger.*

P31 *– I was with my husband for 20 years and…I mean I knew there was something wrong, but I was like ‘is it me, is it him’…I have that knowledge now and so in another relationship that would empower me.*

P37 *– …previously I was just continuously going from, it was almost like a cycle – relationship to relationship to relationship and ending up with the same result…I understand what’s going on and I understand what changes need to be made...*

#### Sharing the story

Three interviewees and all focus groups reported discussing their experiences with someone – i.e. ‘sharing’ their stories – contributing to thriving. This was commonly mentioned within the context of a group setting; many had shared their experiences within the safety of a ‘peer group’ of other women who had also experienced DVA. Through sharing their DVA ‘story’, the abuse no longer defined women, and gave them ownership over the past so they could start healing. It also contributed to a feeling of mutuality - that they were not alone in their experiences.

P27 *– It’s through talking to people that I have accepted it…it’s my story now, it’s not just something where I wasn’t sure what was going on like it’s actually defined, it’s a defined story but it hasn’t defined my personality.*

P18 *– …By talking about it you then realise that actually you are believed and actually that is a big deal because a lot of the time you downplay it and then it makes you see that it has been a big deal.*

#### Social responses

Women in all groups discussed mixed experiences with ‘professionals' they had contact with, including general practitioners, social workers, and the police. Positive interactions included professionals recognising the signs of abuse and supporting women via signposting or providing content for police statements. Negative interactions were commonly due to doctors mis-diagnosing due to missing signs of abuse, or due to criticism from social workers.

Participants in all groups and two interviewees spoke about the need for a society-wide response to DVA, to include: educating professionals on the signs and impact of abuse; ACEs; and teaching in schools about healthy relationships.

P18 – *…I think it should all be widely known about with the professionals you know because there’s so many times when the signs of it are all missed and so many women and children are left in these situations that aren’t picked up on or aren’t felt to be serious. So I think that social workers need more training, doctors need to be more aware of it, psychologists, everywhere really, in schools they need to be able to pick up the signs, I think it’s been missed a lot.*

P4 *–* *[the social worker]* *said ‘how could anyone like you be ever be considered a good mom…with your horrific background’.*

P20 – *My next-door neighbour was a GP and he had to write a court statement, a witness statement, and the judge quoted him saying ‘if you don’t know to look for domestic violence you won’t see it’. And I actually thought that was a really good point…*

#### Forgiveness, acceptance and spirituality

Women reported feeling shame as victims or survivors; they believed they allowed the abuse to happen. Women in two focus groups, and one interviewee, referred to an element of ‘self-forgiveness’ that was required to move forward. However, women in the validation group said that self-forgiveness was irrelevant; the abuse was only ever the fault of the perpetrator as *he chose you, you didn’t choose him* ^(P33)^. They reported that to truly thrive a victim had to have their eyes opened to the fact that the abuse had never been their fault.

All except three participants said that they could never forgive their abuser(s) for what they had done to them or their children, and that this was not necessary to thrive. Instead, women said a level of ‘acceptance’ and ‘letting go’ of the past was required for them to move on, and to thrive.

P25 – *I didn’t forgive myself…because I felt for so long that I allowed it, that’s why I hated myself…you don’t think anyone else is going through it.*

P36 – *I think there’s some things you can forgive and other things you can’t …some things you can just forgive because of the circumstances. But then there’s some things the way people treat people, or things that someone’s done…I just don’t think they deserve forgiveness.*

P3 – *I guess it kind of depends on what you mean by forgiveness because I mean, letting go and forgiving are two different things…because forgiving says it’s okay, what you did to me is okay, and actually it’s not.*

Of those who did wish to forgive, one felt that an element of spirituality helped her through this process, and another mentioned a spiritual cleansing enabled her to forgive in order to ‘move on’:

P17 – *We are spiritual beings all of us. And I’m not talking about religion either, you know it’s about a higher self and bigger picture stuff. And that eventually does aid my healing process and my freedom because eventually…I want to forgive.*

Beyond forgiveness, ‘spirituality’ had also played a part in the recovery for two interviewees and women in two focus groups and was often discussed in relation to therapeutic techniques and coping mechanisms such as *mindfulness* ^(P17)^ or breathing exercises.

Spirituality and religion were usually mentioned alongside each other, but women were keen to separate the two concepts, and these conversations did not always focus on forgiveness but rather hope or support; three women spoke about the role that religious faith played in their recovery via the provision of ‘hope’, or *practical support* ^(P35)^ from people within their religious community.

P31 *– Well I don’t really do religion much these days as I don’t go to church but yeah, I’m a committed Christian and that helped me immensely. I don’t think it’s necessary for everyone, I think it’s a personal thing.*

However, two participants were openly critical of religion, arguing that *religion often supports domestic violence* ^(P31)^.

## Discussion

As with previous studies, women likened thrivership to that of a fluid [20, 31, 32] ‘journey’ [20, 31, 32, 37] with a vulnerability to triggers [37]. Healing from abuse is not prescriptive; rather it is a non-linear process with a variable time-scale [37]. DVA services and professionals should consequently acknowledge the long-term process of overcoming abuse [37] and ensure women are not pressured to recover within a certain time, as this may be detrimental to their well-being.

Thriving featured an element of helping others, as in previous studies [22, 30, 37]. For the minority this took the form of social activism [30], though this was not deemed necessary for thriving. Instead, all had helped others via the transference of new knowledge and tools gained through programmes, to other group members, friends and family. This dedication to supporting other survivors, and the wealth of knowledge thrivers have around DVA, implies they are ideally equipped as programme delivery staff, and for informing service-design.

A ‘reclaiming self’ model of thriving emerged in the current study as in others [19, 20, 30, 32]. In line with previous findings, women reported a re-construction of identity [38]; returning to skills and hobbies [20, 31, 32], work and education [38]; an increase or return of self-confidence [22], self-worth, and “knowing who you are and what you want” [38]. Women experienced empowerment and control over one’s life [20, 31, 32, 37].

Unlike previous literature on thrivership, findings of the current study revealed detailed insight into how an individual’s health differs when ‘thriving’ post-DVA. Wozniak and Allen [20, 31, 32] refer to psychological well-being with a spirituality-based focus of “developing inner peace and serenity” [32], but little beyond this. Some results that emerged were like those by Flasch [37] on long-term recovery; women reported improved health and well-being including healing from mental and physical symptoms of the abuse, recognising the health consequences of the abuse, and the steps they took to promote optimal health (e.g. exercise). Beyond this, women in the current study reported: specific examples of dissipated abuse-related health-conditions (e.g. IBS); being better-equipped with new tools and techniques to manage mental well-being; developing new help-seeking behaviours; and negative experiences due to misdiagnoses and social worker approaches. Further research is recommended into how health-services can be suitably optimised to ensure women can easily and quickly access help when needed.

Thriving was characterised by a social network in line with previous findings [37]; women repaired damaged relationships with family and friends [20, 31], reconnected with those they’d lost, and cut out negative relationships [37]. These networks offered positive social support [37, 39] and ultimately enabled women to re-join their community [20, 31, 32].

As with previous research women reported a more positive outlook: ‘thrivers’ can find joy in everyday circumstances [22] and inspiration in the future [30].

Unlike previous studies, women actively chose not to forgive the abuser as they did not *deserve forgiveness* (P36). This did not prevent them from thriving. In a similar vein, whilst self-forgiveness was thought to be necessary during early recovery, by the ‘thriver’ stage women had acknowledged that the abuse was solely the fault of the abuser, thus self-forgiveness was not necessary; a finding that is also different to those of previous work in this area.

Again, unlike previous studies that reported thriving as featuring a “renewing [of] the spirit” [30] and “healing your soul” [20], most women did not feel that spirituality was necessary to thrive. Similarly, religion, whilst deemed necessary for women in previous studies, was not considered fundamental to thriving for women here.

### The proposed model

**Fig. 1:**
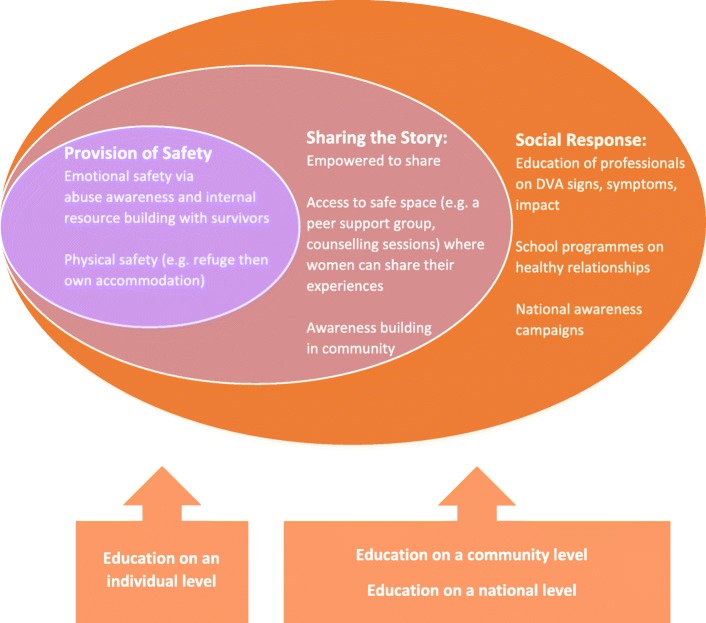
The Thrivership Model with ‘key conditions’ required

The proposed Thrivership Model (Fig. 1) offers a brand-new and unique model that further contributes to our understanding of the theory of thrivership. It illustrates the fundamental requirements for thriving post-DVA, by women who have experienced it.

The proposed model’s key components are: (1) Provision of Safety, (2) Sharing the Story, (3) Social Response (to DVA). ‘Education’ and building awareness around DVA at either an individual, community or national level is required within all three components of the proposed model. All components contribute to ensuring a woman can reach the ‘thriving’ stage of recovery, with each of the features outlined in the findings section, and below.

#### Provision of safety

Women reported – in line with previous findings [20, 31, 32] - that physical safety is required early on for thrivership to occur; physical safety provides the crucial foundation for the thrivership process. However, women experiencing DVA who seek safety continue to experience a range of barriers to accessing refuge spaces, which leaves them vulnerable to further abuse from the perpetrator, at risk of sleeping rough and often left dependent on their social networks for a place to stay [40]. Focus from government policy and funding in these areas is crucial if women are to thrive after DVA.

Women spoke of the need for emotional safety or psychological safety, that could be developed via the acquisition of knowledge and understanding of perpetrator behaviour, common signs of domestic abuse and its impact, and how to recover via the development of internal resources (such as resilience and courage), and assertiveness skills. Education of survivors in this way has not been referred to in previous thrivership research, though empirical evidence has shown that women who have experienced DVA do not always recognise their experiences as abusive and thus need help and support to name the abuse [16]. Additionally, equipping individuals with boundaries and knowledge of early warning signs can help survivors navigate future relationships and break the cycle of abuse [41]. The Freedom Programme – usually delivered as a standalone course - is successful [42] in this area.

As in the current study, previous research has highlighted the impact that relaying ‘positive coping mechanisms’ such as cognitive strategies, relaxation, and coping skills to survivors can have on women’s mental health and well-being [43].

#### Sharing the story

Like previous study findings, “shattering [the] silences” [20, 31] that exist around abuse through sharing one’s experiences with others was reported as key to thriving; ‘sharing the story’ of abuse (see Fig. 1), particularly in the context of support groups, contributes to validation and feelings of mutuality. Crucial to this component is the availability of services (e.g. peer support groups, counsellors) and a culture within which women feel they can share their story free from judgement. The recent draft Domestic Abuse Bill from the UK government set out government plans to raise public awareness of DVA via working with community groups and running media campaigns [44], which may go some way in creating a culture where people who experience DVA feel safe to share their stories.

#### A social response

Women said a ‘Social Response’ is necessary for DVA to be tackled long-term. This component provides a societal ‘backdrop’ for the thrivership process, and education of professionals at victim ‘touch-points’ is crucial to this.

Due to the volume of women who attend healthcare services, primary healthcare professionals are well-placed to identify cases and be a valuable resource for victims via sign-posting to specialist services [45] and  addressing health-related issues to support women towards safety. Previous studies have shown primary healthcare-professionals’ attitudes towards women experiencing DVA are positive, but many continue to only have basic knowledge of the area [46]. Thus, general practitioners and nurses need more comprehensive training on DVA assessment and interventions. Schemes such as IRIS (Identification & Referral to Improve Safety) are already succeeding in this area [47] though funding and sustainability continue to be a concern [47]. We would encourage use of the proposed model in professional teaching programmes for those who come into contact with women who experience DVA, to provide guidance on how women can be supported towards ‘thrivership’ in their recovery journey. The proposed model could also be used to inform the planning and commissioning of services and be used as a framework for support programmes delivered by DVA-specific and charitable organisations so that women receiving the programmes can see the hope for thrivership. Central to meeting the needs of those who experience DVA is that healthcare services acquire good relations with refuges and other non-governmental organisations working on DVA [48], to ensure up-to-date knowledge and pooling of available resources.

Similarly, collaboration between DVA-services and public health bodies could provide educational sessions for those working in social services, to build a culture of understanding around abuse that no longer problematises the mothers [49], whilst still prioritising the children’s safety. Women’s reports of distressing experiences with social workers are in-line with previous research [49]; this must be addressed if women with children are to thrive. Equally, school-based campaigns teaching children about healthy relationships and how to stay safe were highlighted as crucial to preventing the transmission of DVA to future generations and ultimately contributing to the ultimate goal of eliminating DVA altogether. Indeed, the draft Domestic Abuse Bill released in January 2019 has confirmed government plans to make the provision of relationship and sex education compulsory in primary and secondary schools, though this will not be rolled out until September 2020 [44]

This study was undertaken alongside a service evaluation of a women-only service, and thus male ‘victims’ of DVA have not been included. Further research around thrivership to include the viewpoint of male victims, and victims from the LGBTQ+ community may be appropriate, to identify its relevance to these communities and how it can feed into services established to meet their needs. However, it is important to note that DVA continues to be an example of gendered violence in that women predominantly suffer at the hands of men.

#### Limitations

Whilst focus groups were the most suitable methodology for this study, they were not without their disadvantages. It became clear throughout the study that the emotional state of a participant could easily impact their interactions with the group, and the information they shared. Several women who identified as ‘survivors’ had recently got divorced, or been in court, and another informed IH that her perpetrator had emailed her during the focus group; all of these women were emotional during sessions and had more negative views than others. Emotional contagion took place in two groups, particularly when talking about experiences with social workers and past abuse.

Though it is clear from the data what is needed for someone to thrive after DVA, how ‘well’ or quickly a person thrives may also depend on the type, amount of, and quality of external support they receive (e.g. counselling, family).

## Conclusion

Domestic abuse continues to be one of the most widespread human rights abuses and public health problems in the world today [38]. As with previous studies on this topic, women who participated in this study had a wealth of experience and understanding of the impact of DVA [49]. Attending to the words of thrivers in this way offers access to this knowledge pool and facilitates the empathy needed from professionals who work with them and wider society.

The findings of this study provide a new view of thriving post-DVA by women who have lived through it. Beyond that, a new and unique model has been proposed that illustrates what is required for the thrivership process to take place. Qualitative research such as this can consequently contribute to the development of effective intervention strategies; inform policy, legislation and training; and shed new light on implementation of effective services, treatment, resources, and community support for survivors - something that is particularly relevant in this age of patient-centred services.

### Further notes

This research project was conducted alongside a qualitative evaluation of the DVA-service from which the participants for this study were recruited. The Birmingham Freedom Project (BFP) deliver a unique combination of 10–12-week awareness and empowerment programmes for women affected by domestic abuse in the region. The programmes are delivered by two female facilitators who have also experienced abuse themselves. The service offers long-term, ad hoc support to women who attend or have attended their programmes, and free, unlimited access to their programmes; women are able to return to the project as many times as they feel is necessary for them to thrive long-term. It is clear then, that attending the BFP has a direct relationship with the sense of survivorship and thrivership of the participants of this study.

## Data Availability

To protect the identity and safety of the participants, all transcripts and recordings were deleted permanently following data analysis. The authors can be contacted if readers would like further discussions around data and findings.

## Abbreviations

DVA: Domestic violence and abuse
GBV: Gender-based violence
LGBTQ: Lesbian, gay, bisexual, transgender, and queer

## References

[1] United Nations (1993). General assembly: declaration on the elimination of violence against women.

[2] World Health Organization (2012). Understanding and addressing violence against women.

[3] World Health Organization (2017). Violence against women.

[4] Bradbury-Jones C, Appleton JV, Clark M, Paavilaine EA. Profile of Gender-Based Violence Research in Europe: Findings from a Focused Mapping Review and Synthesis. Trauma Violence Abuse. 2017. 10.1177/1524838017719234. Accessed 4 July 2019. 10.1177/1524838017719234 29334031

[5] Council of Europe (2011). Convention on preventing and combating violence against women and domestic violence.

[6] European Union. Convention on preventing and combating violence against women and domestic violence. http://europa.eu/epic/news/2012/20121010_convention_on_preventing_and_combating_violence_against_ women_and_domestic_violence_en.htm. Accessed 25 Apr 2019.

[7] Crown Prosecution Service. Domestic Abuse. https://www.cps.gov.uk/domestic-abuse. Accessed 25 Apr 2019.

[8] National Health Service (NHS) (2017). Domestic violence and Abuse.

[9] Office for National Statistics. Domestic abuse in England and Wales: year ending March 2018. https://www.ons.gov.uk/peoplepopulationandcommunity/crimeandjustice/articles/domesticabusefindingsfromthecrimesurveyforenglandandwales/yearendingmarch2018.

[10] Long J, Harper K, Harvey H (2017). The femicide census: 2017 findings. Annual report on UK femicides 2017.

[11] Office for National Statistics (2016). Intimate personal violence and partner abuse.

[12] García-Moreno C, Jansen HAFM, Ellsberg M, Heise L, Watts CH (2006). Prevalence of intimate partner violence: findings from the WHO multi-country study on women’s health and domestic violence. Lancet.

[13] Campbell JC (2002). Health consequences of intimate partner violence. Lancet..

[14] Walby S (2004). The cost of domestic violence.

[15] Felitti V, Anda R, Nordenberg D, Williamson D, Spitz A, Edwards V, Koss MP, Marks JS (1998). Relationship of childhood abuse and household dysfunction to many of the leading causes of death in adults. Am J Prev Med.

[16] Bradbury-Jones C, Taylor J, Kroll T, Duncan F (2014). Domestic abuse awareness and recognition among primary healthcare professionals and abused women: a qualitative investigation. J Clin Nurs.

[17] Gondolf E, Fisher E (1988). Battered women as survivors.

[18] Moe AM (2007). Silenced voices and structured survival. Violence Against Women.

[19] Merritt-Gray M, Wuest J (1995). Counteracting abuse and breaking free: the process of leaving revealed through women's voices. Health Care Women Int.

[20] Allen K, Wozniak D (2010). The language of healing: women’s voices in healing and recovering from domestic violence. Soc Work Ment Health.

[21] "thrive, v." OED Online. Oxford University Press, June 2019. Web. Accessed 04 July 2019.

[22] Poorman P (2002). Perceptions of thriving by women who have experienced abuse or status-related oppression. Psychol Women Q.

[23] Flach F (1989). Resilience: discovering a new strength at times of stress.

[24] Humphreys J (2003). Resilience in sheltered battered women. Issues Ment Health Nurs.

[25] Tedeschi RG, Calhoun LG (2004). Posttraumatic growth: conceptual foundations and empirical evidence. Psychol Inq.

[26] Paton D, Violanti J, Smith L (2003). Promoting capabilities to manage posttraumatic stress: perspectives on resilience.

[27] Benson PL, Scales PC (2009). The definition and preliminary measurement of thriving in adolescence. J Posit Psychol.

[28] Hall JM, Roman MW, Thomas SP, Travis CB, Powell J, Tennison CR, Moyers K, Shoffner DH, Bolton KM, Broyles T, Martin T, McArthur PM (2009). Thriving as becoming resolute in narratives of women surviving childhood maltreatment. Am J Orthop.

[29] Ickovics J, Park C (1998). Paradigm shift: why a focus on health is important. J Soc Issues.

[30] Taylor J (2004). Moving from surviving to thriving: African American women recovering from intimate male partner abuse. Res Theory Nurs Pract.

[31] Wozniak DF, Allen KN (2012). Ritual and performance in domestic violence healing: from survivor to thriver through rites of passage. Cult Med Psychiatry.

[32] Wozniak DF, Allen KN (2013). Surviving domestic violence: a guide to healing your soul and building your future.

[33] Austin Z, Sutton J (2014). Qualitative research: getting started. Can J Hosp Pharm.

[34] Braun V, Clarke V. Ten fundamentals of qualitative research. In: Braun V, Clarke V, authors. Successful qualitative research: a practical guide for beginners. Los Angeles: Sage; 2013. p. 19–38.

[35] Grant M, Booth A (2009). A typology of reviews: an analysis of 14 review types and associated methodologies. Health Inf Libr J.

[36] Braun V, Clarke V (2006). Using thematic analysis in psychology. Qual Res Psychol.

[37] Flasch P, Murray C, Crowe A (2017). Overcoming abuse: a phenomenological investigation of the journey to recovery from past intimate partner violence. J Interpers Violence.

[38] Crawford E, Liebling-Kalifani H, Hill V (2009). Women’s understanding of the effects of domestic abuse: the impact on their identity, sense of self and resilience. A grounded theory approach. J Int Women's Stud.

[39] Anderson K, Renner L, Danis F (2012). Recovery: resilience and growth in the aftermath of domestic violence. Violence Against Women.

[40] Nowhere to turn: findings from the second year of the no woman turned away project. Women’s aid. 2018. https://1q7dqy2unor827bqjls0c4rn-wpengine.netdna-ssl.com/wp-content/uploads/2018/06/NWTA-2018-FINAL.pdf. Accessed 18 Jan 2019.

[41] Dix L (2018). Cuckoos and recovery: an exploration of responsibility within UK domestic abuse awareness group programmes. J Gend Based Violence.

[42] Abrahams H, Williamson E (2010). Evaluation of the Bristol Freedom Programme.

[43] Nathanson A, Shorey R, Tirone V, Rhatigan D (2012). The prevalence of mental health disorders in a community sample of female victims of intimate partner violence. Partner Abuse.

[44] Transforming the Response to Domestic Abuse: Consultation Response and Draft Bill. HM Government – Home Office. 2019. https://assets.publishing.service.gov.uk/government/uploads/system/uploads/attachment_data/file/772202/CCS1218158068-Web_Accessible.pdf. Accessed 5 Feb 2019.

[45] Evans MA, Feder GS (2015). Help-seeking amongst women survivors of domestic violence: a qualitative study of pathways towards formal and informal support. Health Expect.

[46] Ramsay J, Rutterford C, Gregory A (2012). Domestic violence: knowledge, attitudes, and clinical practice of selected UK primary healthcare clinicians. Br J Gen Pract.

[47] Bradbury-Jones C, Taylor J (2016). Identification and referral to improve safety: local evaluation report for Birmingham South and Central CCG & Birmingham Cross City CCG.

[48] García-Moreno C (2002). Dilemmas and opportunities for an appropriate health-service response to violence against women. Lancet.

[49] Robbins R, Cook K (2018). ‘Don’t even get us started on social workers’: domestic violence, social work and trust—an anecdote from research. Br J Soc Work.

